# Molecular Links between Cancer and Metabolic Diseases: New Perspectives and Therapeutic Strategies for Cancer Prevention and Treatment by Targeting Nutritional Patterns and Metabolic Alterations

**DOI:** 10.3390/cancers15041350

**Published:** 2023-02-20

**Authors:** Mohamed Zaiou, Hamid Morjani

**Affiliations:** 1 Institut Jean-Lamour, Université de Lorraine, UMR 7198 CNRS, 54000 Nancy, France; 2 Unité BioSpecT, EA7506, Université de Reims Champagne Ardenne (URCA), 51096 Reims, France

Cancer-related mortality is reported to be elevated in cases with metabolic dysfunction. Recent evidence from epidemiological, experimental, and clinical studies supports the hypothesis that multiple modifiable risk factors, including diet, obesity, and type 2 diabetes mellitus (T2DM), may be involved in the development and progression of several cancers. Striking metabolic overlap between such cancers and metabolic disorders has sparked great interest in determining the underlying interconnected networks to expand therapeutic options for both types of pathologies. However, mechanisms connecting metabolic dysregulation and the occurrence of some cancers are not yet fully understood.

This editorial summarizes findings from eight articles, which highlight recent advances in understanding the potential link between metabolic disorders and various types of cancer. In patients diagnosed with metabolic disorders, the incidence of gastrointestinal, glandular, and reproductive tract cancers is significantly higher compared to the general population. Moreover, patients with a history or current diagnosis of cancer who are overweight or obese have an increased risk of cancer treatment-related morbidity, recurrence, and decreased quality of life [1].

In this Special Issue, Lossen et al. conducted a retrospective cohort study of 287,357 German outpatients to show that obesity is a significant risk factor for the development of colon, rectal, and liver cancer, partly gender-dependent [2]. The authors conclude that clinical management of overweight patients should include careful and structured risk assessment for developing cancer in order to improve long-term outcomes in these patients. Obesity, hyperlipidemia, insulin resistance (IR), and T2DM have been shown to increase the prevalence of nonalcoholic steatohepatitis (NASH), and complications such as cirrhosis and hepatocellular carcinoma (HCC). In this respect, Gutiérrez-Cuevas et al. comprehensively review the current molecular knowledge of the link between HCC and NASH and highlight the role of epidemiological, metabolic, genetic, and epigenetic alterations involved in these pathologies. Furthermore, the authors shed light on current and future therapeutic strategies for the prevention and treatment of NASH and HCC [3].

Neuraminidase-1 (NEU-1) is thought to be involved in metabolic diseases (NAFLD, obesity, insulin resistance (IR), …) and several cancers (HCC, pancreatic carcinoma, colorectal cancer, breast cancer, …) and could be considered in some cases as a link between these two physiopathological events. In this sense, Toussaint et al. aim to provide a detailed overview of the role of NEU-1 in several metabolic diseases as well as in various cancers and discuss the potential of this enzyme as a pharmacological target for the treatment of some metabolic disease-associated cancers [4].

According to epidemiological and basic studies, alterations in energy metabolism, increased lipid biosynthesis in particular, are emerging as important hallmarks of many forms of liver cancer, including HCC. In addition, the available evidence points to the aberrant expression of enzymes involved in cholesterol metabolism in cancerous tissues [5,6]. For example, the proprotein convertase subtilisin/kexin type 9 (PCSK9) is closely associated with the occurrence and progression of several types of cancer. In this regard, Alannan et al. performed experiments in which they inhibited the function of the enzymes PCSK9 and 3-hydroxy 3-methylglutaryl-Coenzyme A reductase (HMGCR) in hepatoma cell lines using pharmacological inhibitors [7]. The authors show that targeting PCSK9 and HMGCR effectively reduces tumor aggressiveness and disrupts the process of oncogenesis. Based on these findings, they suggest that such enzymes could be considered a therapeutic option for clinical use in liver cancer. Another type of alteration associated with lipid metabolism is the inherited defect in sphigolipid catabolism that leads to lysosomal storage diseases named sphingolipidoses. It has been proven that patients with Gaucher disease, the most common sphingolipidosis, have a high risk of developing malignancies. In the review section of their article, Dubot et al. discuss the potential involvement of lysosphingolipids in cancer and mechanisms underlying the susceptibility of patients with sphingolipid storage diseases leading to the development o cancer [8]. In the second and experimental section, the authors present data on how glucosylsphingosine induces modifications of melanoma cells that may promote tumor progression. Collectively, these studies clearly demonstrate that many metabolic intermediates are involved in cancer processes, supporting a link between metabolic disorders and cancer development.

Resistance to treatment of HCC with sorafenib limits the therapy of choice. The mechanisms underlying this resistance are still unknown. Epigenetic alterations that commonly occur in HCC cells in response to sorafenib link microenvironmental or metabolic changes to cell state transition [9]. The expression of the tumor suppressor receptor, signaling lymphocytic activation molecules family 3 (SLAMF3) is reduced in cancer tissues compared to control tissues. Fouquet et al. show that rescuing the SLAMF3 expression increases the sensitivity of cancer cells to sorafenib and controls metastatic mechanisms in cancer cells by inducing the epithelial-to-mesenchymal transition [10]. In addition to acquired resistance to cancer drugs, tumor angiogenesis could be targeted to improve clinical cancer therapy. Ligustilide, the main component of the essential oil used in traditional Chinese medicine *Angelica sinensis*, has been shown to have inhibitory effects on various types of cancer, including non-small cell lung cancer, osteosarcoma cells, and ovarian cancer cells. In an article by Ma et al., the authors show that ligustilide downregulates VEGFA levels in cancer-associated fibroblasts in prostate cancer via the TLR4-ERK/JNK/p38 signaling pathway and inhibits the promotion of angiogenesis [11].

Obesity, T2DM, and metabolic syndrome are also associated with chronic inflammation, hyperuricemia, and cancer. Hyperuricemia is the result of purine metabolism alteration, which can lead to tumorigenesis. While physiological levels of circulating uric acid are generally considered to have free radical scavenging effects to reduce cancer risk [12], recent data suggest that high levels of serum uric acid may serve as a pro-oxidant factor to generate oxidative stress and inflammatory reactions. However, the mechanisms linking uric acid to carcinogenesis remain unclear. In a review by Allegrini et al., the authors discuss the possible causes of uric acid accumulation or depletion and some of the metabolic and regulatory pathways, with a particular focus on hepatic fructose metabolism [13]. In addition, they shed light on recent findings of the positive and negative effects of fructose on uric acid implication in a variety of signaling pathways that may be implicated in the pathogenesis of diseases such as metabolic syndrome, diabetes, and inflammation, all of which can be linked to some type of cancer.

In summary, the collection of articles presented in this Special Issue offers new insights and a broad view of the potential molecular link between metabolic diseases and cancer, a complex and enthralling research topic with unsolved mysteries. Advances in the study of metabolomics and relevant converging metabolic pathways altered in both cancer and metabolic diseases may offer potential targets to improve cancer diagnosis and possible therapeutic strategies. Furthermore, approaches based on lifestyle changes may be recommended to patients as an adjunct to cancer drug treatment to achieve significant outcomes.
